# A Retrospective Radiographic Study of Metacarpal and Phalangeal Length Proportions: Testing the Golden Ratio, Fibonacci, and Lucas Models

**DOI:** 10.7759/cureus.118834

**Published:** 2026-10-30

**Authors:** Sam Singh, Ka Yan Wong, George Zafiropoulos

**Affiliations:** 1 Trauma and Orthopaedics, Raigmore Hospital, Inverness, GBR; 2 Education, University of South Wales, Cardiff, GBR

**Keywords:** fibonacci sequence, golden ratio, hand anatomy, lucas sequence, metacarpals, phalanges, radiography

## Abstract

Aims

This study tested whether the radiographic lengths of the metacarpals and phalanges conform to five previously proposed proportional models based on the Golden Ratio, the Fibonacci sequence, and the Lucas sequence in adult and paediatric hands.

Methods

A retrospective observational study was performed of posteroanterior hand radiographs obtained at a district general hospital between January 1 and March 9, 2024. Of 467 radiographic examinations screened, 413 hands from 315 patients met the inclusion criteria: 353 adult hands (patients aged 19-95 years) and 60 paediatric hands (aged 6-18 years). Metacarpal and phalangeal lengths were measured on the picture archiving and communication system to 0.1 mm. Five a priori hypotheses were tested: Golden Ratio relationships between adjacent bones, Lucas sequence proportions (1:1.3:2.3:3.6), a 1:1:2 Fibonacci pattern in the little finger, proportional balance between bone segments, and additive sequence behaviour. Each mean ratio or difference was compared with its predicted value using a two-tailed one-sample z-test and its 95% confidence interval (CI), in the whole sample and six subgroups (336 comparisons). A predicted value lying within the 95% CI was taken as not rejected, not as proof of equivalence.

Results

Of 336 comparisons, 324 differed significantly from the predicted values, and 306 remained significant after adjustment (311 at p < 0.001). No consistent Golden Ratio relationships were identified in adjacent bone segments. Subgroup analysis demonstrated isolated approximations, particularly in the proximal-to-middle phalanx ratio of the middle finger in paediatric and adult male groups. The proposed Lucas sequence pattern was largely absent, with only the paediatric ring finger demonstrating a partial correspondence. The little finger did not demonstrate a consistent Fibonacci relationship. Evidence of proportional balance between phalanges was limited to isolated observations, notably in the adult index finger and paediatric middle finger. Additive sequence relationships were similarly restricted to these subgroups.

Conclusion

In this single-centre radiographic sample, metacarpal and phalangeal lengths did not consistently conform to the Golden Ratio, Fibonacci sequence, or Lucas sequence. The few subgroup ratios close to the predicted values were isolated, occurred mostly in the smallest subgroup, and are compatible with chance across multiple comparisons. They should be treated as hypothesis-generating, and confirmation in multicentre and longitudinal samples is required.

## Introduction

The idea that human anatomy might reflect mathematical constants has long fascinated researchers. Huntley [1] and Livio [2] both supported the Golden Ratio (φ ≈ 1.618) theory, and Miller et al. [3] also suggested applying the Fibonacci sequence theory. In the hand, Littler [4] postulated that the spiral geometry of the Golden Ratio underlies the hand’s form. Subsequent investigators have explored whether the relative lengths of the finger bones follow recognisable numerical sequences. Hamilton and Dunsmuir [5] measured finger bone lengths and reported a consistent proportional pattern of approximately 1:1.3:2.3 for distal phalanx (DPh), middle phalanx (MPh), and proximal phalanx (PPh). Littler, expanding on this, stated that the Lucas sequence (a related theory of the Fibonacci sequence) predicts a length progression of 1:1.3:2.3:3.6 when the metacarpal (MC) is included. Miller et al. asserted that Fibonacci numbers predict phalangeal lengths [3]. Other authors, such as Hutchison and Hutchison [6], have drawn parallels to Fibonacci numbers: it has been suggested that the little finger’s three bones might correspond to 1, 1, and 2, echoing the first Fibonacci numbers, indicating that, indeed, a few recent studies claimed that phalangeal lengths conform to Fibonacci relationships. Not all studies agree: in a radiographic study, Park et al. found that the Fibonacci model was not supported, with only the little finger MC-to-PPh ratio approximating φ [7].

Despite these intriguing theories, the evidence for universal mathematical ratios in hand anatomy remains inconclusive. Many of the prior analyses were based on small samples or indirect observations, and the proposed ratios (Golden Ratio, Fibonacci, and Lucas) have not been rigorously validated across a broad population. From a surgeon’s perspective, understanding whether such consistent patterns exist is more than a mathematical curiosity; it could shape our understanding of developmental biology and even how we approach reconstructing digit length or joint placement. If finger bones inherently followed a fixed ratio, it would suggest a fundamental growth principle; if not, it would imply greater variability and the influence of developmental and genetic factors rather than a single ‘design’. If finger bones followed predictable ratios, this could suggest underlying developmental rules, as Gilbert [8] and Hall [9] suggest; mechanical constraints, following Brand and Hollister [10]; or, finally, according to Napier [11] and Susman [12], functional optimisation in skeletal morphology.

This study aimed to test, in a single-centre retrospective radiographic sample, whether MC and phalangeal lengths conform to five proportional models proposed in the literature: (1) a Golden Ratio (φ ≈ 1.618) between adjacent bones; (2) a Lucas sequence ratio of 1:1.3:2.3:3.6 for the DPh, MPh, and PPh and MC; (3) a 1:1:2 Fibonacci pattern in the little finger; (4) proportional balance, in which each bone equals the combined length of the two bones distal to it; and (5) an additive sequence, in which that difference is zero. Each model was tested for every digit in the whole sample and in subgroups defined by age group (adult and paediatric), sex, and side. The primary outcome was whether the predicted value lay within the 95% confidence interval (CI) of the observed mean ratio or difference.

## Materials and methods

A retrospective observational study was performed at District General Hospital. Hand radiographs were retrieved from the institutional picture archiving and communication system (PACS) for the period January 1, 2024, to March 9, 2024. The reporting follows the STROBE (Strengthening the Reporting of Observational Studies in Epidemiology) guidance for observational studies [13]. The hand was chosen because a single posteroanterior (PA) radiograph shows 19 long bones, and because the earlier studies of hand proportions used the same view as described by Hamilton and Dunsmuir [5] and Park et al. [7], allowing direct comparison. Exclusion criteria were non-true PA views, duplicate images of the same hand, poor image quality (e.g., missing parts of bones or images with casts obscuring anatomy), and any X-ray showing acute or healed fractures or deformity of the MC or phalanges. All radiographs were based on the existing calibration of the hospital’s radiology department.

The search returned 467 radiographic examinations, comprising 575 hands. Of these, 162 hands were excluded: 50 were repeat radiographs of a patient already included; 39 showed a fracture; 24 were not a true PA view, or the digits were not extended; 16 did not show every bone completely; 12 showed deformity, erosive arthritis, a congenital anomaly, or a previous amputation; 5 had a cast or metalwork; 5 were not hand radiographs; and 11 had no reason recorded. The remaining 413 hands, from 315 patients, were analysed: 353 adult hands from 257 patients (162 left, 191 right; 158 male, 195 female) and 60 paediatric hands from 58 patients (23 left, 37 right; 30 male, 29 female, 1 not recorded). Ninety-eight patients contributed both hands, and each hand was analysed as a separate observation.

Inclusion criteria include (a) PA radiograph of the hand recorded under the study description ‘XR Hand’ during the study period; (b) all MC and phalanges of the hand fully visible, with both articular surfaces of each bone identifiable; and (c) patients of any age with a recorded age and sex. Exclusion criteria include (a) oblique, lateral, or rotated views that were not a true PA projection; (b) a repeat image of a hand already included; (c) poor image quality, including any bone partly outside the field or obscured by a cast, splint, or dressing; (d) an acute or healed fracture, or deformity, of any MC or phalanx; (e) a cast, Kirschner wire, plate or screws, or a foreign body overlying the bones; (f) arthritis with erosive or destructive change, a congenital anomaly, or a previous amputation; and (g) repeat radiographs of a patient already included.

Patients aged 18 years or younger were classified as paediatric and those aged 19 years or older as adult. The 58 paediatric patients were aged 6 to 18 years (mean 13.7 years, SD 3.4; median 14), and the physes were open in 32 of the 60 paediatric hands. The 257 adult patients were aged 19 to 95 years (mean 51.1 years, SD 18.6; median 53). In paediatric radiographs with open physes, bone length was measured including the epiphysis, from the visible head of the bone (phalanx or MC) to the last visible bone limit at the joint line.

For each hand, we measured the anatomical length of each MC and the PPh, MPh, and DPh of the index, middle, ring, and little fingers. For the thumb, which lacks a MPh, the MC, PPh, and DPh were measured. Measurements were performed with the PACS line tool, from the proximal articular surface to the distal articular surface of each bone, and recorded to the nearest 0.1 mm (Figure 1).

**Figure 1:**
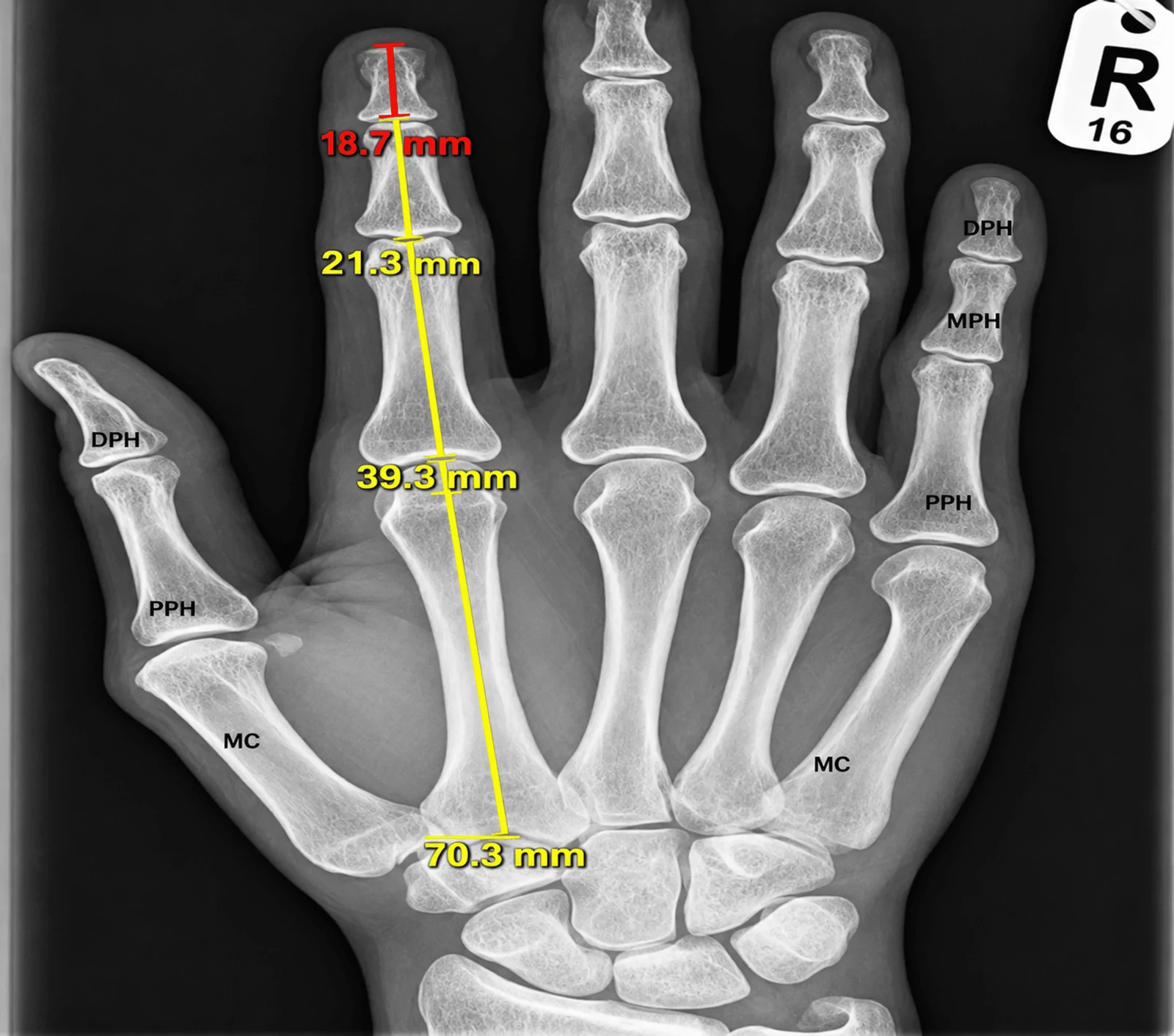
Posteroanterior hand radiograph illustrating the measurement method. DPH: distal phalanx; PPH: proximal phalanx; MC: metacarpal; MPH: middle phalanx

All measurements were made by the second author on the hospital monitor, using the PACS calibration supplied by the radiology department. Length was measured along the long axis of each bone, from the centre of the proximal articular surface to the centre of the distal articular surface. Because the ratio hypotheses compare bones on the same image, any uniform magnification cancels out of the ratios; it affects only the absolute differences in millimetres. 

To ensure measurement reliability, intra-observer reliability was assessed following Popović and Thomas [14]. Forty-eight hands were selected at random, and all 19 bones were remeasured four months later, blind to the first measurements, performed by the first author, giving 912 paired measurements. The mean difference with its 95% CI was used to detect systematic bias, and the 95% limits of agreement (mean difference ± 1.96 SD) were used to describe random measurement error.

Five a priori hypotheses, each taken from the published literature, were tested. The first was that the ratio of adjacent bone lengths equals the Golden Ratio (φ = 1.618) according to Livio [2] and Littler [4]; this was tested for the MC:PPh, PPh:MPh, and MPh:DPh ratios of the index, middle, ring, and little fingers, and for the MC:PPh and PPh:DPh ratios of the thumb. The second was that the DPh, MPh, PPh, and MC follow the Lucas sequence ratio of 1:1.3:2.3:3.6, following Hamilton and Dunsmuir [5] and as reported by Hutchison and Hutchison [6]. Each bone was therefore expressed relative to the DPh, giving predicted values of 1.3 for MPh:DPh, 2.3 for PPh:DPh, and 3.6 for MC:DPh; for the thumb, which has no MPh, the DPh, PPh, and MC were expected to follow the ratio 1:1.3:2.3. The third hypothesis was that the DPh, MPh, and PPh of the little finger follow the ratio 1:1:2, corresponding to the first Fibonacci numbers [4,6], so that MPh:DPh would equal 1 and PPh:DPh would equal 2.

The fourth hypothesis, of proportional balance, was that each bone equals the combined length of the two bones distal to it [15]. This predicts a value of 1 for PPh/(MPh + DPh) and MC/(PPh + MPh) in the fingers, and for MC/(PPh + DPh) in the thumb. The fifth hypothesis expressed the same additive relationship as a difference in millimetres, as in the Fibonacci and Lucas sequences, in which each term is the sum of the two preceding terms, following Santoso et al. [16]. The differences PPh − MPh − DPh and MC − PPh − MPh in the fingers, and MC − PPh − DPh in the thumb, were each predicted to equal zero.

For each hypothesis, digit, and group, the mean observed ratio or difference was calculated with its standard error and 95% CI. The mean was compared with the predicted value using a two-tailed one-sample z-test, with significance at p < 0.05; results at p < 0.001 are reported separately. The 95% CI and the test are two views of the same comparison: a predicted value outside the 95% CI corresponds to p < 0.05. The CI was reported because it shows both whether the predicted value is excluded and how precisely the mean was estimated. With samples of 60 to 413, the normal approximation differs negligibly from the t distribution.

Because each hypothesis predicts equality with a fixed value, a significant result rejects the hypothesis for that digit and group. A non-significant result, with the predicted value inside the 95% CI, was classed as consistent with the hypothesis. This does not demonstrate equivalence; it means only that the data could not exclude the predicted value, and it becomes more likely as the sample shrinks.

Analyses were run on the whole sample (n = 413) and on six subgroups: all adults, adult left, adult right, adult male, adult female, and paediatric hands. These subgroups overlap: the adult subgroups are subsets of the adult group, which is itself part of the whole sample. They are therefore not independent tests. In total, 336 comparisons were made. No correction for multiple comparisons was applied to the primary analysis, because the aim was to identify any departure from the predicted values. Isolated consistent results were therefore interpreted as exploratory.

No a priori sample-size calculation was performed; all eligible radiographs from the study period were included. The achieved precision is shown by the width of the CIs: for adjacent-bone ratios, the 95% CI half-width was about ±0.016 in the larger adult subgroups and about ±0.026 in the paediatric group.

Height, hand dominance, ethnicity, nutritional status, and socioeconomic background are not recorded with radiographs and were not available for analysis. The study was registered as an audit with the hospital’s audit department, discussed verbally, and as it used anonymised, routinely collected radiographs, individual consent or ethical approval was not required according to the audit officer.

## Results

A total of 413 hand radiographs met the inclusion criteria, of which 353 were adult and 60 paediatric. The adult sample comprised 162 left and 191 right hands, from 158 male and 195 female patients (Table 1). For each digit, the ratios and length differences predicted by the five hypotheses were calculated for the whole sample and for six subgroups: all adults, adult left, adult right, adult male, adult female, and paediatric hands. Each mean was compared with its hypothesised value using a two-tailed one-sample z-test, with statistical significance set at p < 0.05. Results that also reached p < 0.001 are identified separately. Because each hypothesis predicts equality with a fixed value, a non-significant result, with a 95% CI that contains the hypothesised value, is the outcome that supports the hypothesis. A significant result indicates that the hypothesis is rejected for that digit and group. Of the 336 comparisons performed, 12 were consistent with the proposed proportions. The remaining 324 differed significantly, with 311 at p < 0.001 (Table 1).

**Table 1:** Participant characteristics.

Group	Number
Adults	353
Paediatric	60
Adults left hands	162
Adults right hands	191
Adults male	158
Adults female	195

Intra-observer variability

Repeated measurement of 48 randomly selected hands showed excellent repeatability. The mean difference between the first and second measurements was 0.03 mm (SD 0.64 mm; 95% CI −0.007 to 0.076 mm). This did not differ significantly from zero (p = 0.10), indicating that there was no systematic measurement bias (Table 2).

**Table 2:** Summary of hypothesis tests, p-values, and significance by method. Each method was tested in all hands (n = 413) and six subgroups (adults, adult left, adult right, adult male, adult female, and paediatric). All p-values come from a two-tailed one-sample z-test comparing the mean ratio (or mean difference) with the hypothesised value. Significance was set at p < 0.05 for every comparison, consistent with the 95% CIs; results significant at p < 0.05 or p < 0.001 are marked. A non-significant p-value with a 95% CI that includes the hypothesised value indicates consistency with the hypothesis. A non-significant p-value indicates no systematic difference between repeat measurements. MC: metacarpal; PPh: proximal phalanx; MPh: middle phalanx; DPh: distal phalanx; CI: confidence interval; ns: not significant

Method	Hypothesised value	Finding (digit, ratio - group)	95% CI	p-value	Significant at p < 0.05?	Significant at p < 0.001?	Marker	Result	Overall conclusion
Intra-observer variability	Mean difference = 0 mm	Repeat measurements (48 hands × 19 bones, n = 912)	−0.007 to 0.076 mm	0.102	No	No	ns	No systematic bias	No systematic bias; measurements repeatable.
Golden Ratio, adjacent bones	φ = 1.618	Middle PPh:MPh - paediatric	1.588-1.641	0.798	No	No	ns	Consistent with φ	Not supported overall: 5 of 98 ratios consistent with φ, mainly middle finger PPh:MPh.
		Middle PPh:MPh - adult right	1.597-1.630	0.555	No	No	ns	Consistent with φ	
		Middle PPh:MPh - adult male	1.586-1.619	0.071	No	No	ns	Consistent with φ	
		Ring PPh:MPh - paediatric	1.596-1.648	0.790	No	No	ns	Consistent with φ	
		Little MC:PPh - paediatric	1.616-1.655	0.083	No	No	ns	Consistent with φ	
Lucas ratio 1:1.3:2.3:3.6	MPh:DPh 1.3; PPh:DPh 2.3; MC:DPh 3.6	Ring PPh:DPh - paediatric	2.276-2.385	0.273	No	No	ns	Consistent with 2.3	Not supported: 1 of 98 ratios consistent; partial match in paediatric ring finger only.
Littler finger Fibonacci 1:1:2	MPh:DPh 1; PPh:DPh 2	PPh:DPh - adult female	1.968-2.017	0.557	No	No	ns	Consistent with 2	Not supported: MPh:DPh > 1 in every group; PPh:DPh ≈ 2 in adult females only.
Proportional balance	Ratio = 1	Index PPh/(MPh + DPh) - adult left	0.993-1.015	0.459	No	No	ns	Consistent with 1	Not supported overall: 3 of 63 ratios ≈ 1 (index in adult left and male; paediatric middle).
		Index PPh/(MPh + DPh) - adult male	0.999-1.025	0.080	No	No	ns	Consistent with 1	
		Middle PPh/(MPh + DPh) - paediatric	0.969-1.002	0.096	No	No	ns	Consistent with 1	
Sequential length differences	Difference = 0 mm	Index PPh − MPh − DPh - adult left	−0.371 to 0.406 mm	0.929	No	No	ns	Consistent with 0	Refuted: 2 of 63 differences ≈ 0, both index PPh – MPh − DPh.
		Index PPh – MPh − DPh - adult male	−0.209 to 0.743 mm	0.272	No	No	ns	Consistent with 0	

Golden Ratio in adjacent bones

When all 413 hands were analysed together, no ratio of adjacent bones approximated the Golden Ratio (φ = 1.618), and every 95% CI excluded φ (Table 2). Subgroup analysis, however, revealed five exceptions. The ratio of the PPh to the MPh (PPh:MPh) of the middle finger was consistent with φ in paediatric hands (95% CI 1.588-1.641; p = 0.80), adult right hands (1.597-1.630; p = 0.56), and adult male hands (1.586-1.619; p = 0.07). In the paediatric group, two further ratios approximated φ: the ring finger PPh:MPh (1.596-1.648; p = 0.79) and the little finger MC-to-PPh ratio (MC:PPh; 1.616-1.655; p = 0.08). The remaining 93 of 98 comparisons differed significantly from φ, 89 of them at p < 0.001. Neither thumb ratio, MC:PPh or PPh:DPh, approached φ in any group. The Golden Ratio hypothesis was therefore not supported, apart from isolated ratios in particular subgroups.

Lucas sequence ratio (1:1.3:2.3:3.6)

The proportion of 1:1.3:2.3:3.6 for the DPh, MPh, PPh, and MC, derived from the work of Hamilton and Dunsmuir [5] and of Hutchison and Hutchison [6], was examined by expressing the length of each bone relative to the DPh (Table 2). For the thumb, the corresponding proportion is 1:1.3:2.3 for the DPh, PPh, and MC. Of the 98 comparisons, 97 differed significantly from their target values, 95 at p < 0.001. The single exception was the paediatric ring finger, in which the PPh:DPh ratio was consistent with the predicted value of 2.3 (95% CI 2.276-2.385; p = 0.27). Even in this finger, however, the complete sequence was not present. The MPh:DPh ratio (1.44) and the MC:DPh ratio (3.27) both differed significantly from their predicted values of 1.3 and 3.6 (p < 0.001). No digit in any group conformed to the full sequence, and the hypothesis was not supported.

Fibonacci sequence according to Littler (1:1:2)

If the phalanges of the little finger followed the first three Fibonacci numbers, the MPh and DPh would be equal in length (MPh:DPh = 1), and the PPh would be twice the length of the DPh (PPh:DPh = 2) (Table 2). The MPh was consistently longer than the DPh. The MPh:DPh ratio ranged from 1.11 to 1.15 across groups and differed significantly from 1 in every case (p < 0.001). The PPh:DPh ratio was consistent with 2 only in adult females (95% CI 1.968-2.017; p = 0.56). It was significantly lower than 2 in adult males (mean 1.94; p < 0.001) and significantly higher in paediatric hands (mean 2.06; p = 0.04). It also differed from 2 in the whole sample and in the remaining adult subgroups (p between <0.001 and 0.04). The data therefore do not support a 1:1:2 pattern.

Proportional balance

The fourth hypothesis proposed that each long bone equals the combined length of the two bones distal to it. This predicts a ratio of 1 for PPh/(MPh + DPh) and MC/(PPh + MPh) in the fingers, and for MC/(PPh + DPh) in the thumb (Table 2). Three of the 63 ratios were consistent with 1, and all three involved the PPh: the index finger in adult left hands (95% CI 0.993-1.015; p = 0.46) and in adult male hands (0.999-1.025; p = 0.08), and the middle finger in paediatric hands (0.969-1.002; p = 0.10). The remaining 60 ratios differed significantly from 1, all at p < 0.001.

The direction of the deviation depended on the digit. The PPh was shorter than the combined MPh and DPh in the middle, ring, and little fingers (ratios 0.91-0.99), but slightly longer in the index finger (1.00-1.05). Similarly, the MC was shorter than the two adjacent bones combined in the thumb, middle, and ring fingers (ratios 0.87-0.94), but longer in the index finger (1.11-1.13) and the little finger (1.05-1.07). No MC ratio approximated 1 in any group. Proportional balance was therefore confined to three PPh ratios in specific subgroups and was not a general feature of the hand.

Sequential length differences

Finally, the additive property of the Fibonacci and Lucas sequences, in which each term equals the sum of the two preceding terms, was tested directly. The differences PPh − MPh − DPh and MC − PPh − MPh were calculated for each finger, and MC − PPh − DPh for the thumb. Each was expected to equal zero. Of the 63 differences, 61 were significantly non-zero, 58 at p < 0.001. Zero lay within the 95% CI only for the index finger PPh − MPh − DPh, in adult left hands (mean 0.02 mm; 95% CI −0.37 to 0.41 mm; p = 0.93) and in adult male hands (mean 0.27 mm; 95% CI −0.21 to 0.74 mm; p = 0.27). The thumb MC was shorter than the sum of the thumb phalanges in every group, by 5.1 to 6.3 mm (p < 0.001). In the remaining digits, the sign of the difference followed the pattern described above. It was negative where the proximal bone was shorter than the two distal bones combined (the phalanges of the middle, ring, and little fingers, and the middle and ring MC). It was positive where the proximal bone was longer (the index finger phalanges, and the index and little MC). The difference for the paediatric middle finger PPh − MPh − DPh only narrowly reached significance (95% CI −1.29 to −0.01 mm; p = 0.048). The additive sequence hypothesis was therefore refuted, except in the index finger of adult left and adult male hands (Table 3).

**Table 3:** Hand bone proportions - overview of hypothesis tests. Every comparison tested at p < 0.05 (two-tailed one-sample z-test); p < 0.001 reported as a second level. Summary of all hypothesis tests: except for intra-observer variability, each analysis was tested in all hands (n = 413) and in six subgroups (adults, adult left, adult right, adult male, adult female, and paediatric). p-values were calculated with a two-tailed one-sample z-test comparing each mean ratio or length difference with its hypothesised value. Significance was set at p < 0.05, and p < 0.001 is reported as a second level. A non-significant result (p ≥ 0.05) means the bones are consistent with the hypothesis; a significant result means the hypothesis is rejected. For intra-observer variability, a non-significant result means there was no systematic difference between repeat measurements. MC: metacarpal; PPh: proximal phalanx; MPh: middle phalanx; DPh: distal phalanx

Analysis	Comparisons tested	Consistent with hypothesis (p ≥ 0.05)	Significant at p < 0.05	Of which p < 0.001	0.001 ≤ p < 0.05	Actual p-values of consistent findings (p ≥ 0.05)	p-value range of rejected comparisons (p < 0.05)	Conclusion
Golden Ratio in adjacent bones	98	5	93	89	4	Middle PPh:MPh: paediatric 0.798, adult right 0.555, adult male 0.071; ring PPh:MPh: paediatric 0.790; little MC:PPh: paediatric 0.083	<0.001 to 0.019	Not supported overall; consistent only for middle PPh:MPh (paediatric, adult right, adult male), ring PPh:MPh, and little MC:PPh (paediatric).
Lucas ratio 1:1.3:2.3:3.6	98	1	97	95	2	Ring PPh:DPh: paediatric 0.273	<0.001 to 0.013	Not supported; only paediatric ring PPh:DPh ≈ 2.3.
Littler finger Fibonacci 1:1:2	14	1	13	9	4	Little PPh:DPh: adult female 0.557	<0.001 to 0.041	Not supported; MPh:DPh > 1 in every group; PPh:DPh ≈ 2 only in adult females.
Proportional balance	63	3	60	60	0	Index PPh/(MPh + DPh): adult left 0.459, adult male 0.080; middle PPh/(MPh + DPh): paediatric 0.096	<0.001 (all)	Not supported overall; consistent only for index PPh/(MPh + DPh) (adult left, adult male) and middle PPh/(MPh + DPh) (paediatric).
Sequential length differences	63	2	61	58	3	Index PPh – MPh − DPh: adult left 0.929, adult male 0.272	<0.001 to 0.048	Refuted; difference ≈ 0 only for index PPh – MPh − DPh (adult left, adult male).

## Discussion

Miller et al. [3] reported that Fibonacci numbers predicted the lengths of the phalanges, and the review by Hutchison and Hutchison [6] concluded that the functional lengths of the little finger phalanges follow a Fibonacci series. Our data do not support a 1:1:2 pattern for anatomical bone lengths. The MPh of the little finger was longer than the DPh in every group (MPh:DPh 1.11-1.15), whereas the pattern requires them to be equal. The PPh was twice the DPh only in adult female hands (n = 195). These findings agree with Park et al. [7], who measured 100 hands radiographically and found that the Fibonacci model was not supported. Proportional balance between the phalanges, proposed by Buryanov and Kotiuk [15], was likewise confined to isolated subgroups.

One likely reason for the disagreement between studies is the definition of length. Choo et al. [17] and Hutchison and Hutchison [6] used functional lengths, between joint centres of rotation or skin creases, whereas this study and Park et al. [7] measured between articular surfaces on radiographs. Park et al. suggested that this difference could account for the discrepancy [7]. Smaller samples such as Choo et al. [17], Suwatyra and Ruwaidiah [18], and Seah et al. [19] may also have produced apparent agreement by chance. Fibonacci-like proportions, where reported, may therefore depend on the method of measurement rather than reflect a property of the bones.

The Golden Ratio (φ), according to Miller et al. [3] and Suwatyra and Ruwaidiah [18], has a near-mythical status in anatomy and art, and people have conjectured that it appears in structures from the human face to the skeletal framework. Littler’s concept of an equiangular spiral in the hand inspired the idea that φ might dictate finger bone proportions. Our data, however, indicate that the Golden Ratio is not a general principle of finger bone anatomy. When all hands were pooled, none of the adjacent bone ratios came close to 1.618. Only after segmenting the data did we find a few φ-like ratios, such as the middle finger PPh:MPh in certain groups. These isolated findings are best regarded as chance agreements. Three of the five φ-consistent ratios occurred in the paediatric group (n = 60), where the CIs were widest and a non-significant result was therefore most likely. The other two involved the middle finger PPh:MPh in adult right (n = 191) and adult male (n = 158) hands, whilst the same ratio differed from φ in all adults combined (n = 353). A developmental explanation, such as relatively shorter MPh during adolescence, is possible but was not tested here. Of note, the paediatric little finger MC:PPh ratio is the same single ratio that Park et al. found close to φ [7]. No evidence supports a universal Golden Ratio template in the hand. For clinicians, this means one should be wary of any claims that hand surgical reconstruction or prosthetic design can rely on φ as a guideline for segment lengths.

The idea of a fixed 1:1.3:2.3 ratio for phalanges originated from radiographic observations by Hamilton and Dunsmuir [5]. This ratio, interestingly, resonates with the Lucas sequence and was expanded by Hutchison and Hutchison [6] to include MC. On the other hand, despite the findings of Paley et al. [20], who found that the Lucas sequence is more prominent in female subjects, the present study’s results provide only limited support for this concept. It was observed that in children (paediatric subgroup), the ring finger’s PPh vs DPh length ratio was ~2.3, which matches the Lucas sequence’s expectation for those two segments. This single agreement, in 60 paediatric hands, is not evidence of a developmental pattern. The other two ratios of the sequence in the same finger differed from their predicted values, and the cross-sectional design cannot show whether proportions change with growth. The paediatric group also combined open and closed physes (32 and 28 hands) across ages 2 to 18 years. We therefore propose, as a hypothesis for longitudinal study, that phalangeal proportions change during growth, and that adult ratios should not be assumed to apply to the immature hand.

Hypotheses 4 and 5 tested whether each bone equals the combined length of the two bones distal to it, the additive rule of the Fibonacci and Lucas sequences. The index finger was the recurring exception: the PPh approximated the combined length of the MPh and DPh in adult left (n = 162) and adult male (n = 158) hands, although not in all adults combined (n = 353). In the middle, ring, and little fingers, the PPh was shorter than the two distal bones combined.

A functional explanation for the index finger is possible but remains a hypothesis. The index finger is the principal partner of the thumb in precision pinch and grip as Napier described [11], and the evolution of human precision grips according to Susman [12] and Marzke [21] has been linked to changes in the proportions of the thumb and fingers. Digit proportions are also shaped by shared developmental pathways rather than by each bone independently. Rolian et al. found that the lengths of the fingers and toes are strongly correlated and appear to have evolved together [22], and Gilbert [8] and Hall [9] suggest that the elements of each digit arise from a common developmental programme. Under this view, phalangeal lengths reflect constraints on growth and function within each digit, not a mathematical rule linking neighbouring bones. This study did not measure function or development, so these explanations require testing, for example by relating segment proportions to pinch biomechanics or by longitudinal measurement during growth.

The number of comparisons must be considered when interpreting the isolated agreements. In total, 336 comparisons were made, across seven overlapping groups. Because each hypothesis predicts equality, a non-significant result is the outcome that appears to support it. A non-significant result becomes more likely when the CI is wide, as in the paediatric group, or when the true value lies close to the predicted one. Among many comparisons, a few will meet this criterion even if no proportional rule exists. The 12 consistent results were scattered across digits, ratios, and groups, and none was reproduced in the whole sample. In no digit did all the ratios of a proposed sequence agree at once. The isolated agreements are therefore compatible with chance and are reported as exploratory, not as evidence of proportional rules in particular subgroups. A formal equivalence test with a prespecified margin would be needed to show that any ratio truly equals a predicted value.

This study has several strengths. Firstly, it is the number of radiographic images studied; it tested five previously published models against the same data, it stated the predicted values in advance, and it reported CIs for every comparison. Measurements were repeatable, with no systematic bias between repeated readings.

The study also has limitations. First, it was a retrospective study from a single institution, so the sample may not represent other populations, and the findings should not be generalised without replication. Second, the design was cross-sectional, so any change in proportions with growth or ageing could not be assessed. Third, 98 patients contributed both hands, and the two hands of one person are not independent; this may have narrowed some CIs. Fourth, the subgroups overlapped and were analysed separately, with no correction for multiple comparisons in the primary analysis. Fifth, the paediatric group was small (60 hands), spanned ages 6 to 18 years, mixed open and closed physes, and was not divided by sex. Sixth, height, hand dominance, ethnicity, and nutritional status were not recorded in the radiographic archive. Nutrition affects longitudinal bone growth in childhood, so it may have influenced the paediatric results in particular. Seventh, two-dimensional radiographs are subject to projection and positioning effects. These are reduced but not removed by the exclusion of non-PA views, and uniform magnification cancels out of the ratios. Measurement between articular surfaces also differs from functional length between joint centres. Eighth, all measurements were made by one observer. Intra-observer agreement was assessed, but inter-observer agreement was not, and the 95% limits of agreement (about ±1.25 mm) are relevant for the shortest bones. Finally, a non-significant result shows only that a predicted value could not be excluded, not that it was confirmed, and the study tested only the models already proposed in the literature.

A longitudinal study might reveal how these ratios change with growth or ageing. For example, the paediatric vs adult differences we observed suggest that certain ratios (like the 2.3 in the ring finger) may only appear during youth and then dissipate; without longitudinal tracking, we infer this developmental effect indirectly. Second, all is based on radiographic measurements, which have some inherent magnification and measurement error. This was mitigated by the use of strict inclusion criteria (true PA views only - calibrated according to the radiology department’s criteria) and demonstration of controlled intra-observer reliability, but minor measurement inaccuracies are possible. Finally, whilst several prominent hypotheses from the literature were covered, the study was not exhaustive in exploring every conceivable numerical pattern. The models already published or postulated in reputable sources were examined, but it remains theoretically possible that other ratios or sequences could emerge in hand anatomy. Given the negative results for these well-known hypotheses, however, any other ‘hidden’ pattern, if it exists, is likely subtle.

## Conclusions

In this single-centre retrospective radiographic study of 413 hands, MC and phalangeal lengths did not consistently follow the Golden Ratio, the Fibonacci sequence, or the Lucas sequence. Of 336 comparisons, 324 differed significantly from the predicted values. The 12 agreements were confined to single ratios in particular subgroups, and no digit conformed to a complete proposed sequence. These isolated agreements are compatible with chance across multiple comparisons and should be regarded as hypotheses for further study, not as proportional rules.

For clinicians, these results suggest that fixed mathematical ratios should not be used to predict digit segment lengths, for example, in reconstruction or prosthetic design. Multicentre studies that record growth, nutrition and functional measures, and longitudinal studies of the growing hand are needed to establish how hand proportions vary and why.
